# Renal Capillary Hemangioma Mimicking Urothelial Carcinoma, A Case Report and Review of the Literature

**DOI:** 10.30699/IJP.14.2.175

**Published:** 2019-06-10

**Authors:** Bita Geramizadeh, Nadereh Shams, Pouya Iranpour, Mohammad Javad Rajabi

**Affiliations:** 1 *MD, Department of Pathology, Shiraz University of Medical Sciences, Shiraz, Iran*; 2 *Transplant Research Center, Shiraz University of Medical Sciences, Shiraz, Iran*; 3 *MD, Kowsar General Hospital, Shiraz, Iran*; 4 *MD, Department of Radiology, Shiraz University of Medical Sciences, Shiraz, Iran*

**Keywords:** Kidney, Capillary hemangioma, urothelial carcinoma

## Abstract

Renal hemangioma is a rare tumor which can be capillary or cavernous. There have been less than 30 renal capillary hemangioma cases reported in the English literature.

Herein we will report a case of renal hemangioma which was detected in a 74-year-old man operated with the impression of urothelial carcinoma of hilum.

## Introduction

Capillary hemangioma is a common benign vascular tumor in the soft tissue and skin, however, its presence in kidney is very unusual and there have been only case reports and very small case series in the English literature. The presence of capillary hemangioma in renal pelvis is an unexpected occurrence and can be misdiagnosed as renal malignancy (1, 2). Majority of the reported cases have been asymptomatic and incidentally discovered during imaging studies or nephrectomy specimens (3). 

In this case report we describe our experience with a 74-year-old gentleman who was operated with the impression of urothelial carcinoma of renal pelvis, but histopathological study of the nephrectomy specimen showed capillary hemangioma of renal pelvis.

## Case Report

A 74-year-old man referred to the urologist for excision of a mass in renal pelvis. 

The mass was detected during sonography of pelvic cavity because of urinary retention secondary to the prostatic hypertrophy. 

No previous history of any significant disease was noted and physical examination was completely normal. The laboratory work up including biochemical and hematological tests including renal function tests were all normal. 

**Figure 1:**
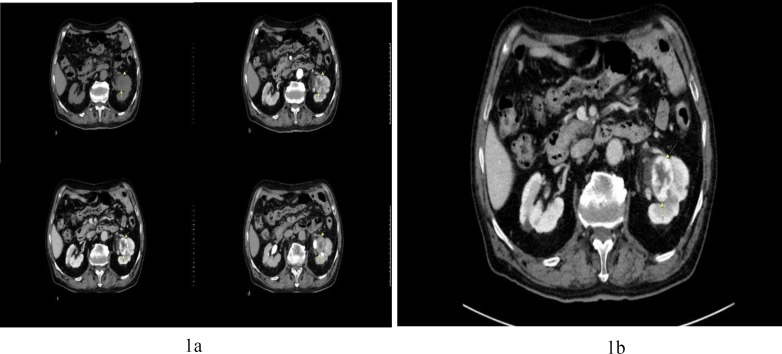
(a) Non-contrast CT scan shows an iso-dense mass. Arterial and portal phase contrast enhanced CT imaging show peripheral nodular enhancement of the lesion with a gradual centripetal fill-in pattern in delayed phase. (b) A solid parapelvic mass with peripheral irregular enhancement in axial contrast CT scan

Ultrasonography showed a well-defined solid-cystic mass in the renal pelvis measuring about 4 cm. The sonographic findings were in favor of a renal tumor in the hilum, thus, CT scan was recommended. The CT scan showed lower limit of size in both kidneys, and small mass in the left renal pelvis (Fig. 1a, b). The mass was heterogeneous with peripheral irregular enhancement, measuring about 4×3.7×2.6 cm. According to the location of the tumor and imaging studies, the main impression was urothelial carcinoma of renal pelvis; thus, the patient was undergone for radical nephrectomy. 

**Figure 2:**
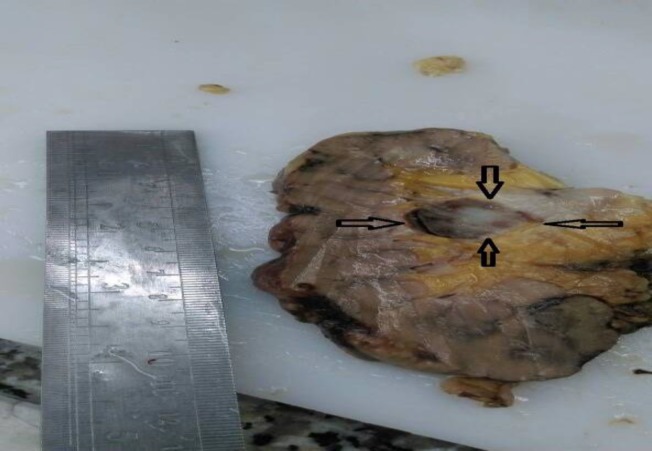
Gross specimen shows a well-defined brownish mass near the hilum (arrow)

The specimen received in the Pathology Department showed small size kidney with fat in-growth and a round well-defined mass in the hilum, measuring about 4 cm in greatest diameter. The mass was mostly solid with white and brown color (Fig. 2).

**Figure 3:**
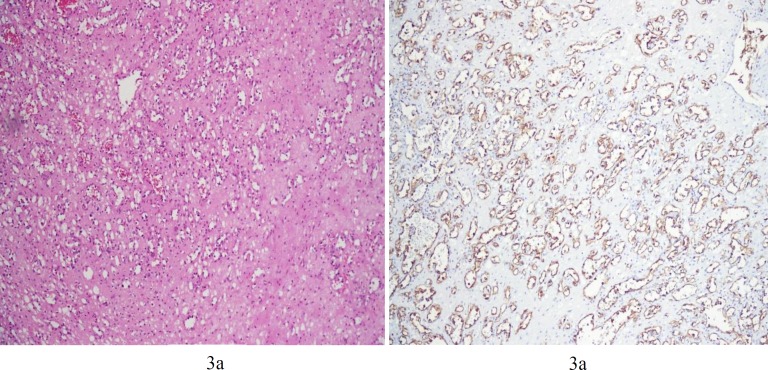
(a) Sections from renal mass show small size capillary type vessels, which are positive with CD31. (b) There is no atypia and no anastomosing channel. (H&E X250)

Multiple sections from the mass showed many capillary-sized vessels which were lined by bland looking endothelium. The endothelial cells showed no atypia and were positive for CD31 and factor-8-antigen. There was no anastomosing channel. (Fig. 3a, b).

The patient was reported with capillary hemangioma of renal pelvis. Follow up for 6 months was unremarkable.

Table-1 shows the details of the reported cases. As the table shows, majority of them occurred in men with the age range of 22-69 years (46.6 ± 12.1).

**Table 1:** Different characteristics of the renal hemangiomas reported since 2000

	Author	Age/Sex	Size(cm)	Accompanied disease	Presentation
**1**	Brown eta l ^1^	64/M	3.5	-	Hematuria
**2**	Brown eta l ^1^	58/M	2	-	Hematuria
**3**	Brown eta l ^1^	40/M	0.2	RCC*	Incidental
**4**	Brown eta l ^1^	69/M	0.4	UC**	Incidental
**5**	Brown eta l ^1^	49/F	Microscopic	RCC	Incidental
**6**	Brown eta l ^1^	60/F	2.1	-	Hematuria
**7**	Brown eta l ^1^	39/M	1	ESRD***	Incidental
**8**	Brown eta l ^1^	40/M	3	-	Incidental
**9**	Mehta et al ^2^	60/M	0.5	RCC	Incidental
**10**	Mehta et al ^2^	39/M	1.8	-	Incidental
**11**	Mehta et al ^2^	63/M	9	Nephrolithiasis	Incidental
**12**	Lee et al ^3^	43/M	2	RCC	Incidental
**13**	Memmedoğlu ^5^	36/M	8	Hypertension	Incidental
**14**	Memmedoğlu ^5^	46/M	9	Hypertension	Incidental
**15**	Neto et al ^6^	40/F	0.5	-	Hematuria
**16**	Nakamura et al ^7^	37/M		-	Hematuria and pain
**17**	Gupta et al ^9^	47/M	2	-	Pain
**18**	Rutherford et al ^10^	22/F	2	-	Hematuria
**19**	Vasquez et al ^11^	62/F	3	-	Incidental
**20**	Leak et al ^12^	39/M	5	Polycythemia	Incidental
**21**	Takeuchi et al ^13^	36/F	8.5	RCC	Incidental
**22**	Buttner et al ^14^	32.6/M	0.6	ESRD	Incidental
**23**	Buttner et al ^14^	69.8/M	0.3	ESRD	Incidental
**24**	Buttner et al ^14^	55/M	0.2	ESRD	Incidental
**25**	Buttner et al ^14^	41.8/M	1.5, 1	RCC&ESRD	Incidental
**26**	Buttner et al ^14^	32/M	0.5, 0.3,0.3,0.6	RCC&ESRD	Incidental
**27**	Buttner et al ^14^	42.3/M	0;3	ESRD	Incidental
**28**	Buttner et al ^14^	45.1/M	1-2.5	ESRD	Incidental
**29**	Buttner et al ^14^	43.5/F	1	RCC&ESRD	Incidental
**30**	Current Case	74/M	3	-	Incidental

* (Renal cell carcinoma,

** Ulcerative colitis,

*** End stage renal disease.

## Discussion

Most of the renal tumors are of epithelial origin and mesenchymal tumors of kidney are rare. Vascular tumors of the kidney are extremely rare and are mainly composed of capillary and cavernous hemangiomas and so called anastomosing hemangioma, hemangio-endothelioma and angiosarcoma. (4) 

There are very rare case reports of renal capillary hemangioma (5). To the best of our knowledge, there have been less than 30 cases of renal capillary hemangioma reported in the English literature since 2000 (6-14). 

Our case with 74 years of age would be the oldest patient. Majority of the previous (80%) cases such as our case have been asymptomatic and were incidentally discovered during work up for other diseases, however, in symptomatic cases, the most common symptom has been hematuria and flank pain (6). The most common location in the kidney was renal medulla and hilum (11). Many of the cases were accompanied by nephrolithiasis and end-stage renal diseases, hypertension and renal cell carcinoma (1-14); however, our case occurred in a healthy 74-year-old male with no significant previous medical history. 

As mentioned above, some of the previously reported renal hemangiomas were detected in nephrectomy specimens of the patients with renal cell carcinoma, and end-stage renal disease. They are mostly unifocal, but there have been only 4 cases of multiple renal hemangiomas detected in the nephrectomy specimen of the patients with end-stage renal disease. (14) Some of the cases such as the current tumor have been detected during imaging studies for other diseases and operated with the impression of renal tumors (mostly renal cell carcinoma and urothelial carcinoma).(6-14) 

Preoperative diagnosis of renal hemangioma is very difficult, because there is no specific radiologic finding in favor of hemangioma. Angiographic findings can be hypo or hypervascular or normal. (3) In our patient, total nephrectomy was done with the preoperative diagnosis of urothelial carcinoma, however, precise evaluation of the CT scan after pathologic diagnosis of renal hemangioma showed the presence of little enhancement which has been sustained into the delayed phase. This finding was indicative of a vascular tumor. (3)

Preoperative diagnosis will be helpful for the decision of nonsurgical treatments such as laser therapy. (7) In the absence of any symptom and complication, no excision is advised and the patient can live without any symptom or complication. (1)

Pathologic diagnosis of renal hemangioma after surgical excision is not challenging. Main differential diagnosis is angiosarcoma which is a highly malignant tumor with cellular atypia, mitosis and necrosis as well as high proliferative index. (2) Benign conditions are mainly vascular malformations occurring in the kidney. Histologically they are consisting of an admixture of abnormally arranged thick and thin-walled vessels, such as malformed veins, venules, arteries, and arterioles, occasionally with associated thrombosis. (1) Another entity in the genitourinary tract is anastomosing hemangioma which is also a solitary and well-demarcated tumor. It differs from capillary hemangioma by scattered large feeding and draining vessels with lobulated appearance as well as scattered areas of stromal edema, and hyalinization as well as collagen deposition. (4)

As a conclusion, renal capillary hemangioma is an extremely rare tumor, mostly diagnosed with no preoperative diagnosis; however, precise evaluation of CT scan can be very helpful for correct diagnosis before unnecessary nephrectomy.
